# Butyrate Feeding Reverses CypD-Related Mitoflash Phenotypes in Mouse Myofibers

**DOI:** 10.3390/ijms22147412

**Published:** 2021-07-10

**Authors:** Ang Li, Xuejun Li, Jianxun Yi, Jianjie Ma, Jingsong Zhou

**Affiliations:** 1 Department of Kinesiology, College of Nursing and Health Innovation, University of Texas at Arlington, Arlington, TX 76010, USA; xuejun.li@uta.edu (X.L.); jianxun.yi@uta.edu (J.Y.); 2 Department of Surgery, Davis Heart and Lung Research Institute, The Ohio State University, Columbus, OH 43210, USA; jianjie.ma@osumc.edu

**Keywords:** butyrate, mPTP, cyclopilin D, amyotrophic lateral sclerosis, jRCaMP1b, supervised inspection of Ca^2+^ transients

## Abstract

Mitoflashes are spontaneous transients of the biosensor mt-cpYFP. In cardiomyocytes, mitoflashes are associated with the cyclophilin D (CypD) mediated opening of mitochondrial permeability transition pore (mPTP), while in skeletal muscle they are considered hallmarks of mitochondrial respiration burst under physiological conditions. Here, we evaluated the potential association between mitoflashes and the mPTP opening at different CypD levels and phosphorylation status by generating three CypD derived fusion constructs with a red shifted, pH stable Ca^2+^ sensor jRCaMP1b. We observed perinuclear mitochondrial Ca^2+^ efflux accompanying mitoflashes in CypD and CypDS42A (a phosphor-resistant mutation at Serine 42) overexpressed myofibers but not the control myofibers expressing the mitochondria-targeting sequence of CypD (CypDN30). Assisted by a newly developed analysis program, we identified shorter, more frequent mitoflash activities occurring over larger areas in CypD and CypDS42A overexpressed myofibers than the control CypDN30 myofibers. These observations provide an association between the elevated CypD expression and increased mitoflash activities in hindlimb muscles in an amyotrophic lateral sclerosis (ALS) mouse model previously observed. More importantly, feeding the mice with sodium butyrate reversed the CypD-associated mitoflash phenotypes and protected against ectopic upregulation of CypD, unveiling a novel molecular mechanism underlying butyrate mediated alleviation of ALS progression in the mouse model.

## 1. Introduction

Mitoflash events are spontaneous fluorescent transients of the mitochondrial targeted, circularly permuted yellow fluorescent protein (mt-cpYFP) [1]. In cardiomyocytes, the frequency of mitoflash events decreased by two-thirds under anoxia and rebound to 1.9-fold of normoxia levels 5 min after reoxygenation, implying an association between these events and a reperfusion induced burst of reactive oxygen species (ROS) [1]. In skeletal muscle, mitoflash events have been associated with ROS production, mitochondrial respiration acceleration under tetanic electrical field stimulation, and heat-induced hypermetabolism [2,3]. More recently, new evidence emerged to link mitoflash events with pathological elevations of mitochondrial ROS levels in myofibers after denervation or during the progression of neuromuscular degeneration of amyotrophic lateral sclerosis (ALS) [4,5].

Although the connections between mitoflash events and ROS production have been reported in multiple studies, it is debated whether these events are authentic indicators of superoxide burst or merely representing mitochondrial matrix alkalization [1,6,7,8]. Yet there may exist a common ground that can be substantiated by both sides, as both matrix alkalization and generation of ROS can be associated with the opening of the mitochondrial permeability transition pore (mPTP) [9,10,11,12,13,14]. mPTP is a nonselective mega-channel, permitting solute (<1.5 kDa) exchange between the mitochondrial matrix and the outside milieu [15,16,17,18,19]. The opening of mPTP is sensitive to a series factors including Ca^2+^, pH, and cyclophilin (Cyp) D [15,16,17,18,19]. CypD is a mitochondrial matrix protein recently reported to physically interact with oligomycin sensitivity-conferring protein (OSCP) within ATP synthase (complex V of OXPHOS) [20], which is believed to be a molecular component of mPTP within the inner mitochondrial membrane (IMM) [21,22,23,24]. A scenario linking matrix alkalization to the mPTP opening was proposed more than two decades ago: Ca^2+^ influx into mitochondria depolarizes IMM, triggering a compensatory effect of the respiratory chain to pump out H^+^; once the matrix alkalization passes a threshold, mPTP opens and leads to briefly collapsed IMM potential, Ca^2+^ efflux, and matrix acidification [9,25,26]. Conversely, the mPTP opening alters ionic strength, which can disrupt the electrostatic interaction between cytochrome c (Cyto C) and cardiolipin in the mitochondrial intermembrane space. Cyto C is required for the activity of ubiquinol-cytochrome c oxidoreductase (complex III of OXPHOS) [11]. The blockage of complex III activity enhances ROS production by increasing the accumulation of the one-electron donor ubisemiquinone [12,13,14]. The mPTP opening also seems to induce conformation changes in NADH ubiquinone oxidoreductase (complex I of OXPHOS), leading to elevated ROS production [27]. 

The connection between mitoflash events and the mPTP opening is supported by experimental results in cardiomyocytes [1]. However, in the wild type mouse flexor digitorum brevis (FDB) myofibers mitoflash activities are not significantly affected by the addition of cyclosporin A (CsA, an inhibitor of the CypD-mediated mPTP opening) or knocking out CypD [2,3]. However, denervated myofibers or myofibers from a mouse model of ALS (hSOD1^G93A^) exhibited notably elevated mitoflash activities, which were attenuated by the application of CsA [4,5]. These discrepancies may be explained by the difference of mitochondrial function under physiological and pathological conditions. More specifically, under physiological conditions, a stochastic mPTP opening is a rare event, hence mitoflash activities predominantly correlate with transient mitochondrial respiration accelerations. Denervation or neuromuscular degenerative disease changes mitochondrial Ca^2+^ load ([Ca^2+^]_mito_), ROS levels, and CypD expression in myofibers [4,5]. Thus, the pathologically elevated mitoflash activities can result from a CypD-dependent mPTP opening. Supportive evidence of this hypothesis suggests that mitoflash activities under physiological conditions stochastically occur in individual mitochondrion or several mitochondria in close proximity [2,3], while those under pathological conditions can occur over large areas of a mitochondrial network in a synchronized manner, sometimes even throughout the whole diameter of the myofiber [4,5]. Thus, it is believed that mitoflash activity is strongly associated with the opening of mPTP under pathological conditions in skeletal muscle. 

To further evaluate the potential connections between CypD and mitoflash phenotypes, we created three fusion constructs. The first is the full length of the mouse CypD, inserted upstream of a red shifted, pH stable Ca^2+^ sensor jRCaMP1b [28]. By electroporation of this construct into FDB myofibers expressing mt-cpYFP, we not only overexpress CypD, but also target jRCaMP1b to the mitochondrial matrix to monitor [Ca^2+^]_mito_ changes during mitoflash events. CypD function has been reported to be regulated by a series of post-translational modifications, including phosphorylation, acetylation, oxidation, S-nitrosylation, S-palmitoylation, and S-glutathionylation [29,30,31,32,33,34,35,36,37,38,39,40,41]. Among them, phosphorylation of serine 42 (S42) is reported to increase in mitochondrial uniporter (MCU) knockout mice [33]. Additionally, using CypD knockout mouse embryonic fibroblasts, ectopic expression of CypD carrying phospho-resistant mutation at residue 42 (S to A) exhibits lower mPTP opening probability in response to Ca^2+^ stimulation [33]. Thus, we created a second construct inserting CypDS42A upstream of jRCaMP1b to assess the impact of phospho-resistant CypD on mitoflash phenotypes. The third construct only has the mitochondria-targeting sequence of CypD fused with jRCaMP1b, which serves as a negative control [42,43,44]. 

Last but not least, our previous study showed that butyrate feeding significantly alleviated the disease progression in hSOD1^G93A^ mice [45]. This mutation is known to upregulate CypD expression and change mitoflash activities [5]. Here, we also evaluated whether butyrate treatment affects mitoflash phenotypes related to CypD expression levels.

## 2. Results

### 2.1. Fusion Constructs of CypD Derived Peptide and jRCaMP1b Exhibit Mitochondrial Localization and Respond to Ca^2+^ Stimulation

**Figure 1:**
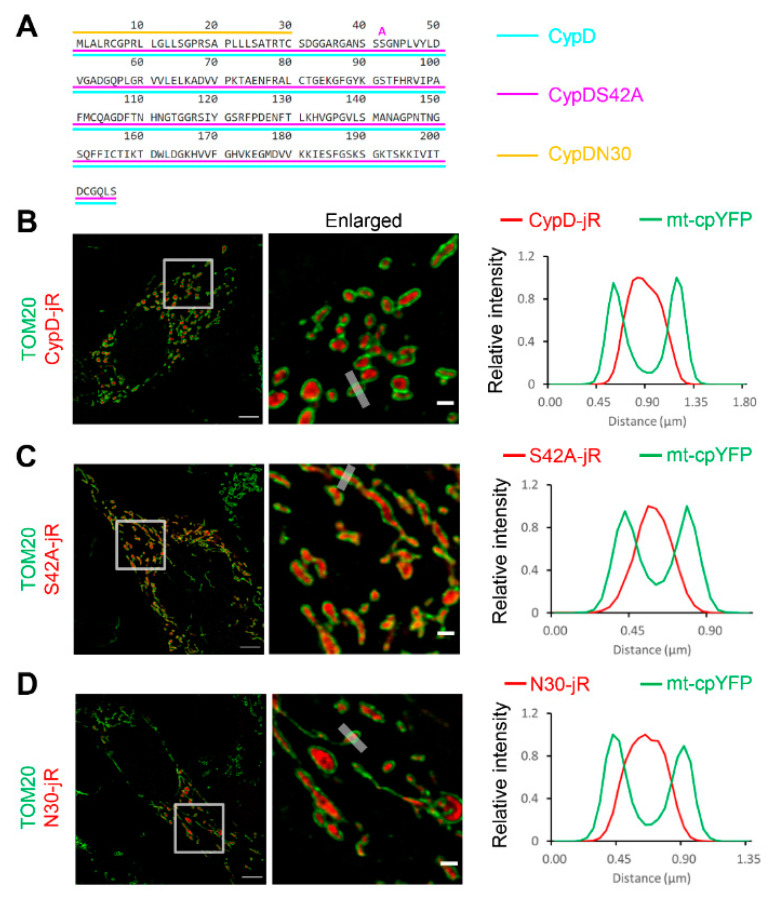
CypD derived fusion constructs exhibit mitochondrial localization. (**A**) Schematic drawing of the three peptide sequences inserted upstream of the genetically encoded Ca^2+^ indicator jRCaMP1b. (**B**–**D**) CypD-jRCaMP1b (shortened as CypD-jR), CypDS42A-jRCaMP1b (shortened as S42A-jR) and CypDN30-jRCaMP1b (shortened as N30-jR) all exhibited predominant localization inside the mitochondria of C2C12 cells. Immunostaining of TOM20 (green) highlights the outer mitochondrial membrane. Boxes highlight enlarged area. Grey strips in the enlarged image highlight areas for relative intensity (*F* − *F_min_*)/(*F_max_* − *F_min_*) profiling on the right. Scale bars: 5 µm, 1 µm.

**Figure 2:**
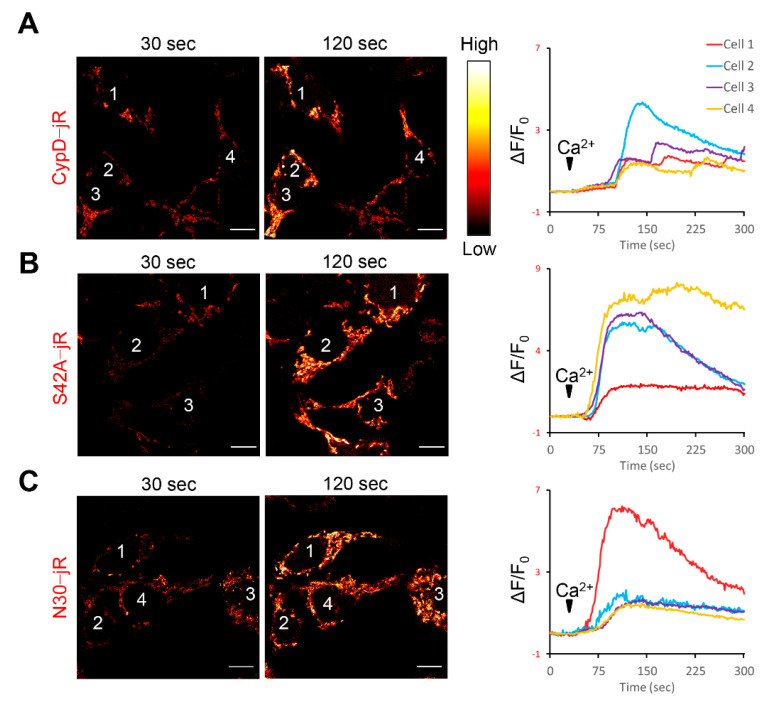
CypD derived constructs responded to Ca^2+^ stimulation in RyR_2_ expressing cells. (**A**–**C**) Most HEK-tet-RyR_2_ cells transfected with either CypD-jRCaMP1b, CypDS42A-jRCaMP1b or CypDN30-jRCaMP1b for 2 days and induced to express RyR_2_ for 18 hrs exhibited increased intensity (red hot pseudo-color) upon external Ca^2+^ stimulation. Corresponding jRCaMP1b traces of cells numbered in the images are shown on the right. Ca^2+^ concentration in external solution increased from 0 mM to 2 mM at 30 sec (arrowheads). Scale bars, 10 μm.

We generated three fusion constructs by combining mouse CypD derived peptides with jRCaMP1b. These peptides included full length mouse CypD (without stop codon), a phospho-resistant mutation at serine 42 (S42A) [33], and the mitochondria targeting sequence of CypD (coding sequence of the 30 amino acids at the N-terminus, as a negative control) [44] (Figure 1A). All three constructs were predominantly localized in the mitochondria after transfecting mammalian cell lines (Figure 1B–D). The representative images and kymographic measurements were from transfected C2C12 cells due to their flat morphology (the mitochondrial network spreads out mainly in XY plane, facilitating kymographic measurement). Next, we examined whether the jRCaMP1b in the fusion constructs could still respond to Ca^2+^ level changes, because C2C12 cells poorly express ryanodine receptors. We transfected the four constructs into HEK-tet-RyR_2_ cells, a specialized cell line highly expressing RyR_2_ after tetracycline treatment. These HEK cells exhibit a store overload induced Ca^2+^ release (SOICR) upon elevation of extracellular Ca^2+^, resulting in Ca^2+^ influx into the mitochondria [46,47]. The jRCaMP1b intensity notably increased in response to external Ca^2+^ stimulation (0 mM to 2 mM) for all three fusion constructs (Figure 2 and Videos S1–S3), confirming that the fusion setup did not undermine the functionality of jRCaMP1b. 

### 2.2. Observation of Mitoflash Associated Mitochondrial Ca^2+^ Efflux in the Perinuclear Region of Myofibers

**Figure 3:**
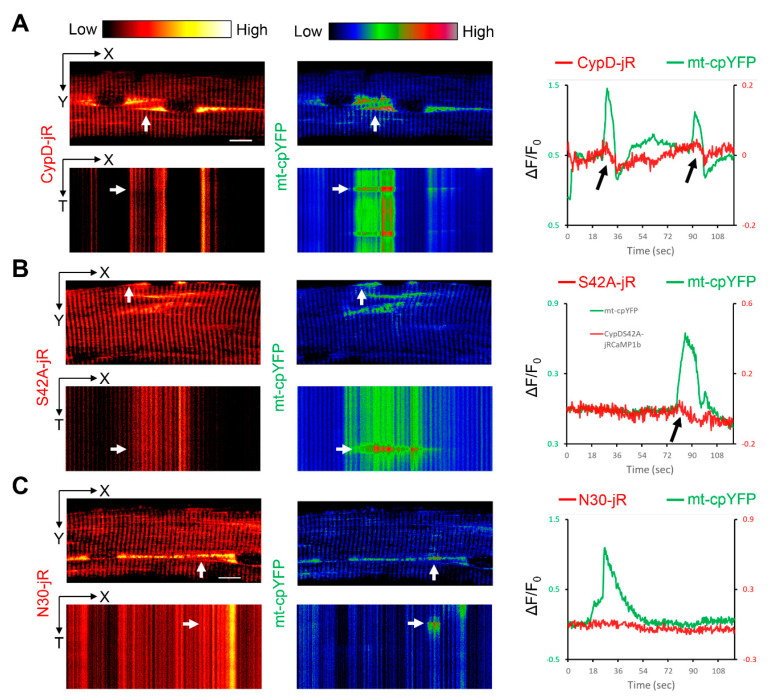
Observation of mitoflash associated mitochondrial Ca^2+^ efflux in the perinuclear region of myofibers. (**A**) Representative time-lapse recording of mouse FDB myofibers comparing CypD-jRCaMP1b (red hot pseudo-color) and mt-cpYFP (rainbow RGB pseudo-color) signals in the perinuclear mitochondria. X-Y view of the myofiber is collapsed along the time axis (T) and X-T view is collapsed along the Y axis. Relative intensity changes (ΔF/F_0_) are measured at the perinuclear area (white arrows). Black arrows denote drop of jRCaMP1b intensity accompanying mitoflash events. (**B**) Representative recording of mouse FDB myofibers comparing CypDS42A-jRCaMP1b and mt-cpYFP signals in perinuclear mitochondria. Black arrows denote drop of jRCaMP1b intensity accompanying mitoflash events. (**C**) Representative recording of mouse FDB myofibers comparing CypDN30-jRCaMP1b and mt-cpYFP in perinuclear mitochondria. Scale bars, 10 µm.

Next, we examined mitochondrial Ca^2+^ dynamics accompanying mitoflash events by co-electroporating the above jRCaMP1b based constructs together with mt-cpYFP into mouse FDB myofibers. For the three constructs tested, most mitoflash events in interfibrillar mitochondria were accompanied by no or subtle changes of [Ca^2+^]_mito_ levels (a total of 91 interfibrillar regions analyzed in myofibers from 18 mice, see Figure S1A–C and Spreadsheet S1). This result may be supportive of the idea that mitoflash events are independent of CypD-mediated mPTP opening [2]. However, this can be due to the relatively low mitochondrial density at the interfibrillar region and the highly stringent [Ca^2+^]_mito_ regulation in myofibers, causing interfibrillar [Ca^2+^]_mito_ changes beyond the detection limitation of jRCaMP1b. Consistent with this idea, the application of pulsatory electric field stimulation only marginally increased the intensity of mitochondria-targeted jRCaMP1b, while the intensity of CaSiR-1 (representing cytosolic Ca^2+^ changes) increased tremendously (Figure S1D). The Ca^2+^ affinity of jRCaMP1b (K_d_ = 0.71 µM) and CaSiR-1 (K_d_ = 0.58 µM) are similar (*n* = 4) [28,48]. More importantly, in the perinuclear mitochondria, in which mitochondrial density is higher, we observed mitoflash events accompanied by drops of jRCaMP1b signals, especially in CypD-jRCaMP1b overexpressing myofibers (8 out of 31 perinuclear regions analyzed in myofibers from 5 mice, also see Spreadsheet S1, Figure 3A, and Video S4). One case was observed in CypDS42A-jRCaMP1b overexpressed myofibers (1 out of 19 perinuclear regions analyzed in myofibers from 4 mice, see Spreadsheet S1, Figure 3B, and Video S5). No such phenotype was observed in CypDN30-jRCaMP1b overexpressed myofibers (0 out of 23 perinuclear regions analyzed in myofibers from 7 mice, see Spreadsheet S1, Figure 3C, and Video S6). These observations indicate that under pathological conditions like CypD overexpression, at least part of the mitoflash events is indicative of an mPTP opening with transient mitochondrial Ca^2+^ efflux. We did not observe a mitoflash-associated decrease of jRCaMP1b signals in interfibrillar mitochondria of CypD-jRCaMP1b overexpressed myofibers, this may be due to lower mitochondrial density and the detection limitation of JRCaMP1b. 

### 2.3. Reversal of CypD-Related Mitoflash Phenotypes in Myofibers by Butyrate Treatment

Next, we systematically compared the properties of mitoflash events under different conditions using a newly developed analysis program called a supervised inspection of Ca^2+^ transients (SICT) [49]. Although SICT was originally used for automatic identification of Ca^2+^ transients from time-lapse recordings, it is capable of analyzing any type of transient signals, such as mitoflash events. There are several advantages of using SICT: (1) SICT automatically identifies transient signals in time-lapse movies with low signal-to-noise ratios, which dramatically reduces manual labor and human bias. (2) It also provides an event inspection interface that allows humanized quality control when needed. (3) SICT uses the moving average for F_0_ (baseline), rather than a fixed F_0_, which minimizes the photobleaching effect and allows more precise segmentation of the transients. Here, we slightly modified the original codes to quantify mitoflash properties, including frequency, 2D area ratio (unique pixel area of mitoflash events divided by the area of mitochondria in the recording, in other words, for events with partially or fully overlapped area, the area was only quantified once), amplitude, half rise time (HRT), half decay time (HDT), and full width half maximum (FWHM).

**Figure 4:**
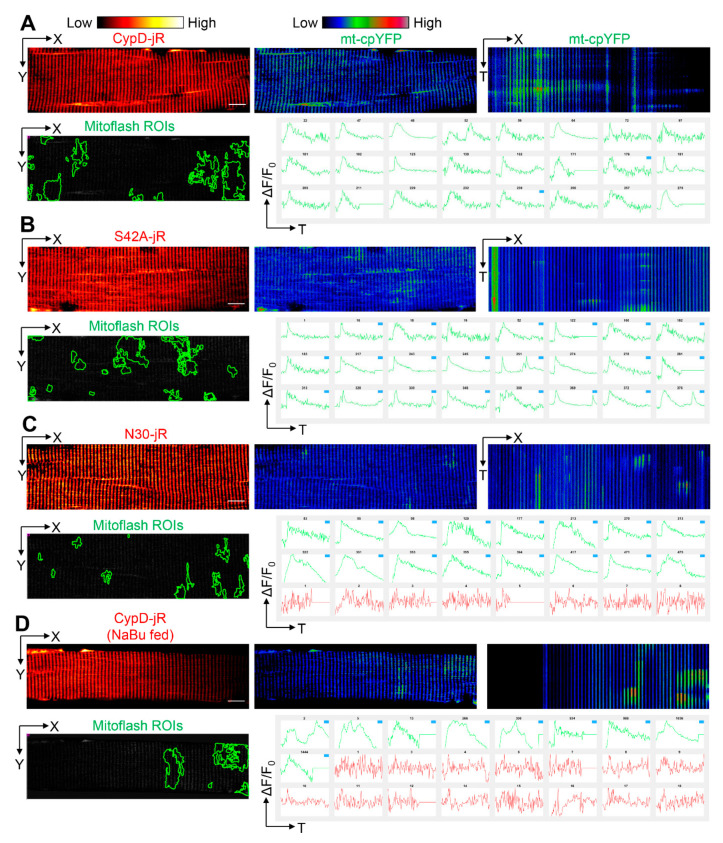
Automatic generation and visual inspection of mitoflash ROIs using SICT. (**A**) Upper panel: representative time-lapse recording of mouse FDB myofibers electroporated with CypD-jRCaMP1b (red hot pseudo-color) and mt-cpYFP (rainbow RGB pseudo-color). Lower panel: boundaries of mitoflash ROIs automatically detected by SICT and confirmed by the viewer are highlighted in green. Top 24 traces corresponding to these ROIs in the SICT visual inspection interface are shown on the right. (**B**) Representative time-lapse recording of mouse FDB myofibers electroporated with CypDS42A-jRCaMP1b and mt-cpYFP used for SICT. (**C**) Representative time-lapse recording of mouse FDB myofibers electroporated with CypDN30jRCaMP1b and mt-cpYFP. Red traces are excluded by the viewer from downstream quantification because they do not have a transient-like profile. (**D**) Representative time-lapse recording of mouse FDB myofibers electroporated with CypD-jRCaMP1b and mt-cpYFP. This group of mice were fed with 2% sodium butyrate (NaBu) in drinking water after electroporation until imaging (7 days). Red traces are excluded by the viewer from downstream quantification. Scale bars, 10 μm.

**Figure 5:**
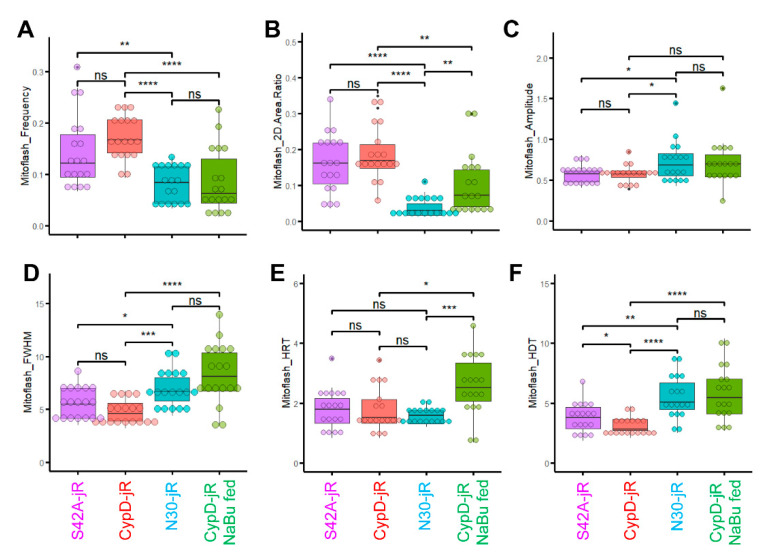
Mitoflash properties quantified by SICT. (**A**) Frequency of mitoflash events, with Hz as the unit. (**B**) 2D area ratio of mitoflash events (the ratio between the unique pixel area of the ROIs and the unique pixel area of the mitochondria in the recording). (**C**) Averaged amplitude of mitoflash events (ΔF/F_0_ at the peak). (**D**) Averaged full width half maximum (FWHM) of mitoflash events (FWHM refers to the timespan from when signal intensity rose to 50% of the peak amplitude to when the signal dropped to 50% of the peak amplitude), with sec as the unit. (**E**) Averaged half rise time of mitoflash events (the timespan in which signal intensity rose from 50% of the peak amplitude to the peak amplitude), with sec as the unit. (**F**) Averaged half decay time of mitoflash events (the timespan in which signal intensity drops from the peak amplitude to 50% of the peak amplitude). * *p* < 0.05; ** *p* < 0.01; *** *p* < 0.001; **** *p* < 0.0001; ns: not significant (Student’s *t*-test).

Through this analytical approach, we identified more frequent mitoflash events in CypD-jRCaMP1b overexpressed myofibers (18 myofibers from 5 mice) compared with CypDN30-jRCaMP1b overexpressed myofibers (18 myofibers from 4 mice, see Figure 4A, C, X-T view, Figure 5A, Videos S7 and S9, and Spreadsheet S2). The 2D area ratio was also significantly higher, implicating that mitoflash events occurred more broadly in the mitochondrial network (Figure 4A, C mitoflash ROIs, Figure 5B, Videos S7 and S9, and Spreadsheet S2). However, the averaged amplitude was slightly smaller (Figure 5C). The averaged FWHM and HDT were significantly shorter (Figure 4A, C X-T view, Figure 5D,F, Videos S7 and S9, and Spreadsheet S2), while the averaged HRT was comparable (Figure 5E and Spreadsheet S2). Mitoflash events in CypDS42A-jRCaMP1b overexpressed myofibers (18 fibers from 4 mice) exhibited similar properties to those in CypD-jRCaMP1b overexpressed myofibers and significantly differed from those in CypDN30-jRCaMP1b overexpressed myofibers (Figure 4B and Figure 5, Videos S8, and Spreadsheet S2). Although CypDS42A is a phospho-resistant mutation of CypD reported to decrease mPTP opening probability, the original study was conducted in a mouse embryonic fibroblast cell line depleted of endogenous CypD [33]. Thus, the similar mitoflash properties in CypD-jRCaMP1b and CypDS42A-jRCaMP1b overexpressed myofibers may be because the increased CypD level overwhelmed the impact of phosphorylation modification at serine 42. These observations support that the molecular mechanisms driving mitoflashes can be different at different CypD levels.

In our previous studies, we identified sodium butyrate (NaBu) as a potent reagent to slow the progression of ALS in hSOD1^G93A^ mice, whose hindlimb muscles exhibited increased expression of CypD and changed mitoflash activities [5,45]. Here, we also examined whether feeding the mice with NaBu (2% in water) changes the CypD related mitoflash phenotypes. Indeed, the myofibers from CypD-jRCaMP1b overexpressed mice fed with NaBu after electroporation (18 fibers from 5 mice) exhibited mitoflash properties closer to those of CypDN30-jRCaMP1b overexpressed counterparts (Figure 4D and Figure 5, Video S10, and Spreadsheet S2). More specifically, the mitoflash frequency and area were significantly decreased compared with those CypD-jRCaMP1b overexpressed myofibers from mice not fed with NaBu (Figure 4D and Figure 5A,B), while the averaged FWHM, HRT, and HDT were all prolonged (Figure 4D and Figure 5D–F). The extension of the FWHM, HRT, and HDT may be due to the promotion of mitochondrial respiration by NaBu [50,51], while the decreased frequency and area of mitoflash events may result from an inhibitory effect on the CypD-related mPTP opening. Since NaBu itself is a histone deacetylase inhibitor [52], this inhibitory effect may be due to an epigenetic mechanism. 

### 2.4. Butyrate Feeding Protects against Ectopic Upregulation of CypD in Myofibers from hSOD1^G93A^ Mice

**Figure 6:**
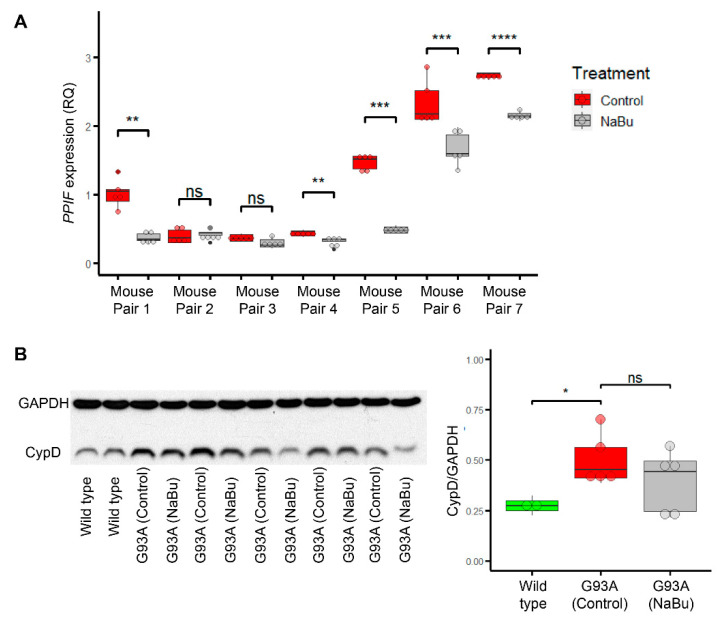
Butyrate feeding protects against ectopic upregulation of CypD in myofibers from hSOD1^G93A^ mice. (**A**) Gender matched littermates of 3 month old ALS mice (hSOD1^G93A^) were divided into control and NaBu (2% in drinking water) fed group. TA muscles were collected for RT-qPCR after 4 weeks. Relative quantification (RQ) of *PPIF* (the gene encoding CypD) was calculated by ΔΔCt method against housekeeping gene *GAPDH* (five technical replicates). ** *p* < 0.01; *** *p* < 0.001; **** *p* < 0.0001; ns: not significant (Student’s *t*-test). (**B**) Western blot results for CypD protein in TA muscles from two wild-type mice and five pairs of hSOD1^G93A^ mice (littermates born at the same time) with or without 2% NaBu in drinking water for 4 weeks from 3 months of age. * *p* < 0.05; ns: not significant.

Our previous study showed butyrate feeding significantly alleviates the ALS disease progression of hSOD1G93A mice through improving gut integrity [45]. Since it is known that butyrate also acts as an HDAC inhibitor [53], here, we examined whether the butyrate treatment in the hSOD1G93A mice have an impact on CypD expression in skeletal muscle of the same ALS mouse model. Several pairs of gender-matched hSOD1^G93A^ mice littermates were fed with or without 2% NaBu in drinking water starting at the age of ALS disease onset (3 months) for 4 weeks. Then, the tibialis anterior (TA) muscle samples were collected for qPCR to assess whether NaBu regulates CypD at transcription level (encoded by gene *PPIF*). In five out of these seven pairs, the NaBu fed littermates exhibited significant downregulation of *PPIF* (Figure 6A and Spreadsheet S3). For the other two pairs that did not show significant difference, the control littermate also had relatively low *PPIF* compared to other groups. Thus, NaBu exhibited a protective effect against ectopic upregulation of *PPIF* during the disease progression of hSOD1^G93A^ mice. We also examined the CypD protein level (normalized to GAPDH) in TA muscles from five pairs of hSOD1^G93A^ mice (littermates of the same batch) with or without NaBu feeding for 4 weeks from 3 months of age, as well as two wild-type mice (Figure 6B, Figure S2 and Spreadsheet S4). The butyrate fed mice exhibited a general trend of lower CypD protein level, although not statistically significant. This lack of significance may be attributable to several factors, not limited to gender differences, variations in the daily consumption rate of NaBu water, disease onset time, disease progression speed, etc. 

## 3. Discussion

In this study, we used genetically encoded, mitochondria-targeted Ca^2+^ indicators instead of chemical dyes, such as Rhod-2, for [Ca^2+^]_mito_ measurement [1,3]. Rhod-2 is proposed to leak out of the mitochondrial matrix during mPTP opening, complicating the interpretation of intensity change [1]. Another advantage of using jRCaMP1b is its small pKa value, making it relatively stable to pH changes [28]. Although most of the mitoflash events we analyzed were accompanied by subtle or no changes of jRCaMP1b signal, we did observe multiple cases of mitoflash-associated drops of jRCaMP1b intensity in the perinuclear regions of CypD-jRCaMP1b overexpressed myofibers. Those mitoflash-associated transient mitochondrial Ca^2+^ effluxes further support that mitoflash events may reflect or correlate to CypD-mediated mPTP opening in skeletal muscle. The results were also supportive of the idea that CypD-mediated mPTP opening can occur in myofibers under certain pathological conditions. It provides an explanation as to why mitoflash activities in myofibers after denervation or during ALS progression were CsA sensitive [4,5]. In the future, with the development of more sensitive, pH stable and red-shifted Ca^2+^ biosensors, we believe more conclusive answers can be acquired regarding mPTP opening related Ca^2+^ dynamic changes in interfibrillar mitochondria under pathological conditions. 

In previous studies the application of an mPTP activator (atractyloside) did not significantly alter mitoflash properties in skeletal muscle [2,3] but increased mitoflash frequency in cardiomyocytes [1]. Atractyloside works through an adenine nucleotide translocator (ANT) located on the inner mitochondrial membrane [54,55], which is considered a part of the mPTP pore in the classical mPTP model [56,57]. More recent studies show that various mouse tissues express ANT isoforms at different levels and the expression of one isoform can increase in compensation for the loss of other isoforms [58]. The classical model was challenged by recent discoveries indicating ATP synthase as the pore forming unit of mPTP on the inner mitochondrial membrane [21,24]. Although the arguments about the molecular basis of mPTP are not fully resolved yet, it is possible that the molecular compositions of mPTP are different between cardiac and skeletal muscle, explaining why actractyloside affects mitoflash activities in cardiomyocytes but not myofibers. 

In perinuclear mitochondria, we also observed several cases of mitoflash events accompanied by a slight elevation of jRCaMP1b intensity (Spreadsheet S1) since Ca^2+^ is known to stimulate the activity of multiple dehydrogenases related to tricarboxylic acid (TCA) cycle, as well as complexes I, III, IV, and V of the respiratory chain [10,59,60,61,62]. This observation supports the view that certain mitoflash events result from acceleration of mitochondrial respiration [2]. 

Butyrate is a short-chain fatty acid derived from microbial fermentation of dietary fibers in the colon [63]. A series of beneficial effects of butyrate at intestinal and extra-intestinal levels have been reported in the last two decades [52]. It is a well-known inhibitor of HDAC, enabling epigenetic regulation of multiple genes [53]. However, it is a source of energy that can be converted to acetyl-CoA through β-oxidation in mitochondria, entering the TCA cycle to reduce NAD^+^ to NADH and providing substrate for oxidative phosphorylation [51]. In our previous studies, we discovered that feeding ALS hSOD1^G93A^ mice with 2% NaBu improved gut integrity and prolonged their life span [45]. In this study, we went forward to examine the potential direct impact of NaBu on myofibers. NaBu protects against ectopically increased expression of CypD, either through HDAC-related epigenetic regulation or a feedback mechanism due to alleviated mitochondrial oxidative stress. This can help explain the decreased mitoflash frequency and area in CypD-jRCaMP1b overexpressed myofibers in NaBu fed mice. Efforts are needed to explore the detailed mechanism of this regulation in future studies. Conversely, we observed prolonged mitoflash FWHM, HDT, and HRT in CypD-jRCaMP1b overexpressed myofibers in NaBu fed mice, suggesting the molecular mechanisms driving these mitoflash events may have changed. This could be due to the promotion of mitochondrial respiration by the energy source role of NaBu. Consistent with this, the mitoflash events in our control myofibers (CypDN30-jRCaMP1b) have lengthier mitoflash FWHM and HDT than in CypD-jRCaMP1b overexpressed myofibers. Thus, the temporal profile of mitoflash events may serve as an indicator to distinguish the molecular mechanisms driving mitoflash activities. 

## 4. Materials and Methods

### 4.1. Animal

All animal experiments were carried out in strict accordance with the recommendations in the *Guide for the Care and Use of Laboratory Animals* of the National Institutes of Health. The protocol on the usage of mice was approved by the Institutional Animal Care and Use Committee of the University of Texas at Arlington (A19.001, approval date: 20 September 2018). Wild-type mice used in this study were of B6SJL background. The ALS transgenic mouse model (hSOD1^G93A^) with genetic background of B6SJL was originally generated by Drs. Deng and Siddique’s group at Northwestern University and deposited to the Jackson Lab as B6SJL-Tg (SOD1*G93A). For the butyrate feeding experiment, wild-type mice electroporated with CypD fusion constructs were fed with 2% NaBu (Sigma ARK2161, St. Louis, MO, USA) dissolved in drinking water from the day of electroporation and lasting a week until myofiber imaging. To characterize butyrate induced gene expression changes in the TA muscles of G93A mice, the animals were fed with 2% NaBu from the age of 3 months (at the ALS disease onset) [5,64,65] and lasting for 4 weeks.

### 4.2. Plasmid Construction

pCAG-mt-cpYFP is a gift from Dr. Heping Chen’s lab. To generate CypD-jRCaMP1b, jRCaMP1b was PCR amplified from pGP-CMV-NES-jRCaMP1b (Addgene #63136) using the following primers: TTTGGTACCGATCTCGCAACAATGGTCGAC and GGTTTTGAATTCCTACTTCGCTGTCATCATTTGTAC. The PCR product was inserted between KpnI and EcoRI to replace the mRFP-GFP cassette in a CypD-mRFP-GFP plasmid, cloned previously. To generate CypDN30-jRCaMP1b, CypDN30 was PCR amplified using primers: TGCGGCCGCCATGCTAGCGCTGCGTTGC and TCTGGTACCGCAGGTACGGGTCGCGGA. The PCR product was inserted into the NotI and KpnI sites upstream of jRCaMP1b. To generate CypDS42A-jRCaMP1b, point mutation was achieved with the primer set: CGAACTCTTCCGCCGGGAACCCGCTCGT and ACGAGCGGGTTCCCGGCGGAAGAGTTCG following Stratagene QuickChange site-directed mutagenesis protocol. All constructs were confirmed by Sanger sequencing at UTA Life Science Core Facility.

### 4.3. Time-lapse and Static Imaging of Cultured Cells

HEK293 cells with inducible expression of RyR_2_ (HEK-tet-RyR_2_) were kindly provided by Dr. Wayne Chen. Both C2C12 cells and HEK-tet-RyR_2_ cells were cultured in DMEM + 10% FBS at 37°C, 5% CO_2_. Transfection of plasmids was conducted using Lipofectamine 3000 regent (Thermo Fisher, L3000008, Dallas, TX, USA) and cells were imaged or fixed for immunostaining 2 days post transfection. For HEK-tet-RyR_2_ cells, RyR2 expression was induced by applying tetracycline hydrochloride (0.1 μg/mL) for 18 h. C2C12 cells were fixed with 4% paraformaldehyde and stained with TOM20 rabbit polyclonal antibody (Proteintech 11802-1-AP, Rosemont, IL, USA). The secondary antibody used was anti-rabbit-Alexa Fluor 488 (Thermo Fisher). All imaging experiments were conducted using a Leica TC SP8 confocal microscope. The time-lapse recordings of HEK-tet-RyR_2_ cells were captured at 1.5 sec/frame. jRCaMP1b was excited with a 561 nm laser and the emission light was collected at 575–625 nm.

### 4.4. Electroporation, Dissection, and Time-lapse Imaging of Myofibers

The procedure of electroporating plasmids into FDB myofibers was described previously [66]. Briefly, the anesthetized mice were injected with 15 µL of 2 mg/mL hyaluronidase (Sigma H4272) dissolved in sterile saline at the ventral side of the hind paws through a 31-gauge insulin syringe. A total of 1.5 h later, 25 µg of plasmid DNA was injected into the same sites. Twelve min later, two electrodes (stainless steel acupuncture needles) were placed at the starting lines of the paw and toes, separated by 9 mm. An amount of 20 pulses of 100 V/cm and 20 ms were applied at 1 Hz (ECM 830, BTX Harvard Apparatus, Holliston, MA, USA). Seven days later, the animal was euthanized by CO_2_ inhalation. FDB muscle was dissected out and digested with 2 mg/mL collagenase (Sigma C0130) in 0 mM Ca^2+^ Ringer solution for 2 hrs. The digested muscle was washed with 0 mM Ca^2+^ Ringer solution twice and then transferred to 2.5 mM Ca^2+^ Ringer solution. After a 30 min wait, the muscle was triturated into single myofibers for imaging. All imaging experiments were conducted using Leica TC SP8 confocal microscope. The time-lapse recordings in Figure 3 and Figure 4 were captured at 0.36 sec/frame for a total length of 2 min. The XT scan in Figure S1D was captured at 0.005 sec/line for a total length of 3 s. Electric field stimulation was applied as 10 ms, 20V pulses using a Grass S48 stimulator and two gold-plated stainless steel acupuncture needles as electrodes (4 mm apart). For imaging cytosolic Ca^2+^ dynamics, chemical dye CaSiR-1 AM (Goryo chemical GC403, Sapporo, Hokkaido, Japan) was mixed with Pluornic F-127 and applied to dissociated myofibers at 5 µM final concentration in 2.5 mM Ca^2+^ Ringer solution for 50 min at room temperature. The myofibers were washed 4–5 times with Ringer solution before imaging. jRCaMP1b was excited with a 561 nm laser and the emission light was collected at 575–625 nm. Mt-cpYFP was excited with a 405 and 488 laser and the emission light was collected at 500–550 nm. CaSiR-1 AM was excited with a 633 nm laser and the emission light was collected at 645–695 nm. 

### 4.5. SICT Based Analysis of Mitoflash Properties

The MATLAB based program SICT was originally developed by Dr. Alexander J. Groffen’s group at VU University [49]. For our analysis, the source codes were modified to adapt files generated by Leica software. Rise time and decay time were modified to half rise time (the timespan in which signal intensity rises from 50% of the peak amplitude to the peak amplitude) and half decay time (the timespan in which signal intensity drops from the peak amplitude to 50% of the peak amplitude). The boundaries of regions of interests (ROIs) generated by the program and confirmed by users for parameter calculations were highlighted by green lines in the visual inspection interface. Below is a simplified description of the procedure: the raw file of the mitoflash channel was imported into the preprocessing program of SICT for automatic generation of ROIs; the ROIs confirmed by the user in the visual inspection interface were imported into the postprocessing program to quantify a series of properties including frequency, 2D area ratio (the ratio between the unique pixel area of the ROIs and the unique pixel area of mitochondria in the recording, segmentation and measurement of the mitochondrial area were done in ImageJ), averaged amplitude, averaged full width half maximum (FWHM refers to the timespan from when signal intensity rose to 50% of the peak amplitude to when the signal dropped to 50% of the peak amplitude), averaged half rise time (the timespan in which signal intensity rose from 50% of the peak amplitude to the peak amplitude), averaged half decay time (the timespan in which signal intensity dropped from the peak amplitude to 50% of the peak amplitude). Please refer to Spreadsheet S2 for details. 

### 4.6. RNA Extraction, Reverse Transcription, and qPCR

Tibialis anterior (TA) muscles from butyrate feed (2% in drinking water for 4 weeks) or control ALS mice (hSOD1^G93A^) were dissected, snap-frozen in liquid nitrogen and stored at −80 °C. For RNA extraction, TA muscles were immersed in RNA*later* ICE (Thermo Fisher AM7030) for more than 24 h and then transferred to Trizol reagent for homogenization using FastPrep-24 (MP Biomedicals 6004500, Santa Ana, CA, USA). The homogenate was transferred into tubes containing 5 PRIME Phase Lock Gel (Quantabio 2302830, Beverly, MA, USA), and 1/10 volume of 1-bromo-3-chloroporpane (Sigma) was added. The samples were shaken vigorously for 12 s and centrifuged for 15 min at 16,000× *g*. Afterwards, the upper phase was transferred to another Eppendorf tube and isopropanol (1/2 volume of the initial homogenate) was added for precipitation overnight at −20 °C. Next day, after 15 min centrifugation at 16,000× *g* the precipitate was washed briefly with 75% ethanol. After another 5 min centrifugation, the ethanol was removed. The precipitate was briefly dried and resuspended in RNase free water. RNA concentration was measured with Quantus Fluorometer (Promega E6150, Madison, WI, USA) and reverse transcription was done using Promega GoScript Reverse Transcription system. Maxima SYBR Green/ROX qPCR Master Mix (Thermo Fisher) was used for qPCR reactions. The qPCR primers were: CTTCCACAGGGTGATCCCAG (*PPIF* forward); ACTGAGAGCCATTGGTGTTGG (*PPIF* reverse); AGGTCGGTGTGAACGGATTTG (*GAPDH* forward); TGTAGACCATGTAGTTGAGGTCA (*GAPDH* reverse). Ct value measurement was done using StepOnePlus Real-Time PCR system (Thermo Fisher). 

### 4.7. Western Blot

Proteins were extracted by grinding in 10 volumes of RIPA buffer containing protease inhibitors (Thermo Fisher Scientific), resolved by SDS-PAGE, and transferred to PVDF membrane with Bio-Rad semidry transfer cell (1703940, Plano, TX, USA). The primary antibodies were CypD (Abcam ab110324, Waltham, MA, USA) and GAPDH (CST 5174, Danvers, MA, USA). Protein bands were detected with Bio-Rad Clarity ECL kit and ChemiDoc Imaging system. Signal strengths and backgrounds were measured using ImageJ software.

### 4.8. Statistics

Box-and-dot plots and performance of statistical tests were generated with ggplot2 package of R. We used Student’s t-test instead of ANOVA in Figure 5 because we preferred to separately highlight the two observations: (1) the rescue effect of butyrate treatment; (2) the similarity between mitoflash phenotypes in CypDS42A-jRCaMP1b and CypD-jRCaMP1b overexpressed myofibers. In Figure 6A, we used Student’s t-test in a pair-by-pair manner versus pooling all samples together. This is because the seven pairs of hSOD1^G93A^ mice were not perfect biological replicates. It is known that the rate of disease progression has a certain degree of variability across batch and gender. The seven pairs of mice were not born at the same time, four of them were male and three of them were female. Additionally, the extracted RNAs were stored for different periods of time before reverse transcription. Thus, it is more meaningful to compare littermates of the same gender, born at the same time, and with RNA extracted at the same time. In contrast, the five pairs of hSOD1^G93A^ mice in Figure 6B were littermates born at the same time, rendering them slightly better biological replicates to be pooled together for statistical analysis, although factors like gender differences, variations in NaBu consumption rate, disease onset time, and disease progression speed may still confound the results. 

## 5. Conclusions

In this study, we generated three CypD derived fusion constructs with Ca^2+^ biosensor jRCaMP1b to compare mitoflash properties and mitochondrial Ca^2+^ dynamics in myofibers. We observed cases of mitochondrial Ca^2+^ efflux in the perinuclear mitochondria in myofibers with overexpression of CypD or CypDS42A, but none in control myofibers expressing nonfunctional CypDN30. These observations support a connection between mitoflashes and mPTP opening at high CypD levels in myofibers. Furthermore, we discovered that feeding mice with butyrate, a short-chain fatty acid derived from microbial fermentation, reversed CypD-related mitoflash phenotypes in myofibers, indicating a protective role of butyrate in skeletal muscle mitochondria. In the ALS mouse model hSOD1^G93A^, butyrate feeding protected against the ectopic increase of CypD, further suggesting that butyrate mediated-mitochondrial protection in skeletal muscle may contribute to the alleviation of disease progression in hSOD1^G93A^ mice. Whether this protection effect is achieved through the HDAC inhibition or a feedback mechanism due to improved mitochondrial oxidative stress is worth further investigation.

## Data Availability

The data presented in this study are available in Supplementary Materials.

## Supplementary Materials

The following are available online at https://www.mdpi.com/article/10.3390/ijms22147412/s1.
