# Supplementary material for: Butyrate Feeding Reverses CypD-Related Mitoflash Phenotypes in Mouse Myofibers
Source: Int J Mol Sci. 2021 Jul 10;22(14):7412. doi: 10.3390/ijms22147412 (PMC8304904; doi:10.3390/ijms22147412)
Supplement: Supplementary file 1 [file ijms-22-07412-s001.zip › Supplementary Material description.pdf]

Figure S1: Subtle changes of mitochondrial $Ca^{2+}$ accompanying mitoflash events in interfibrillar region of myofibers. (A) Representative time-lapse recording of mouse FDB myofibers comparing CypD-jRCaMP1b (red hot) and mt-cpYFP (rainbow RGB) signals in interfibrillar mitochondria. X-Y view of the myofiber is collapsed along the time axis (T) and X-T view is collapsed along the Y axis. Relative intensity changes ($\Delta F/F_0$) are measured at the interfibrillar area denoted by white arrows. (B) Representative time-lapse recording of mouse FDB myofibers comparing CypDS42A-jRCaMP1b and mt-cpYFP signals in interfibrillar mitochondria. (C) Representative time-lapse recording of mouse FDB myofibers comparing CypDN30-jRCaMP1b and mt-cpYFP signals in interfibrillar mitochondria. (D) Pulsatory electric field stimulation of myofibers expressing CypDN30-jRCaMP1b and stained with $Ca^{2+}$ sensitive dye CaSiR-1 AM. Arrows denote the cytosolic and mitochondrial $Ca^{2+}$ transients. Scale bars, 10 μm.

Figure S2: Original Western blot image for Figure 6B. Sample identities (left-right) are available in Spreadsheet S4 (top-down).

Video S1: CypD-jRCaMP1b response to $Ca^{2+}$ stimulation in HEK-tet-$RyR_2$ cells.

Video S2: CypDS42A-jRCaMP1b response to $Ca^{2+}$ stimulation in HEK-tet-$RyR_2$ cells.

Video S3: CypDN30-jRCaMP1b response to $Ca^{2+}$ stimulation in HEK-tet-$RyR_2$ cells.

Video S4: A recording of perinuclear mitoflashes in CypD-jRCaMP1b overexpressed myofiber.

Video S5: A recording of perinuclear mitoflashes in CypDS42A-jRCaMP1b overexpressed myofiber.

Video S6: A recording of perinuclear mitoflashes in CypDN30-jRCaMP1b overexpressed myofiber.

Video S7: A recording of mitoflash events in CypD-jRCaMP1b overexpressed myofiber.

Video S8: A recording of mitoflash events in CypDS42A-jRCaMP1b overexpressed myofiber.

Video S9: A recording of mitoflash events in CypDN30-jRCaMP1b overexpressed myofiber.

Video S10: A recording of mitoflash events in CypD-jRCaMP1b overexpressed myofiber from NaBu fed mice.

Spreadsheet S1: Categorization of jRCaMP1b responses accompanying mitoflash events and mouse information.

Spreadsheet S2: Mitoflash properties quantified by SICT and mouse information.

Spreadsheet S3: RQ values of qPCR results and mouse information.

Spreadsheet S4: Western blot results and mouse information.
