# Supplementary figures and images for: Butyrate Feeding Reverses CypD-Related Mitoflash Phenotypes in Mouse Myofibers
Source: Int J Mol Sci. 2021 Jul 10;22(14):7412. doi: 10.3390/ijms22147412 (PMC8304904; doi:10.3390/ijms22147412)

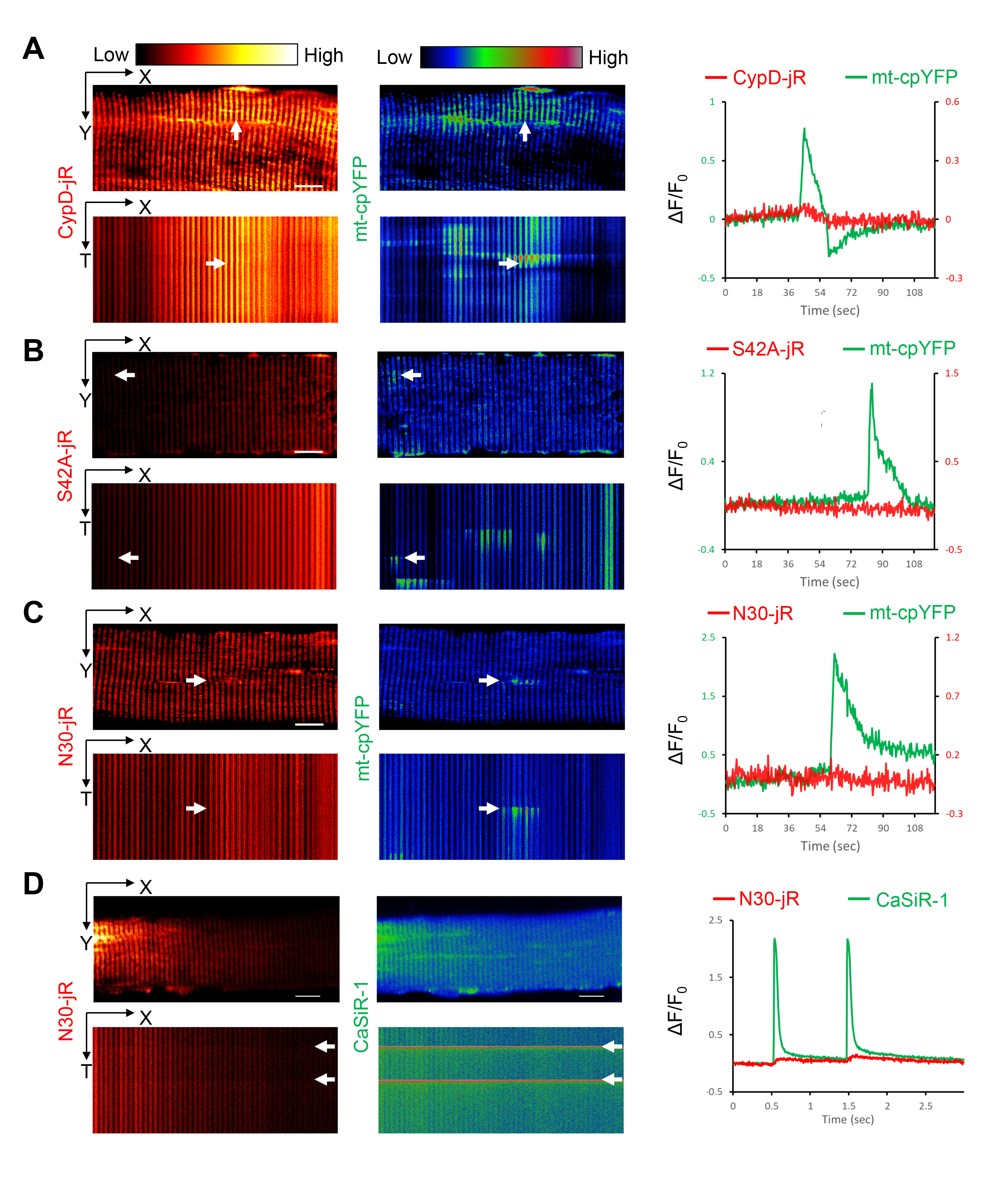

Supplement: Supplementary file 1 [file ijms-22-07412-s001.zip › Figure S1.png]

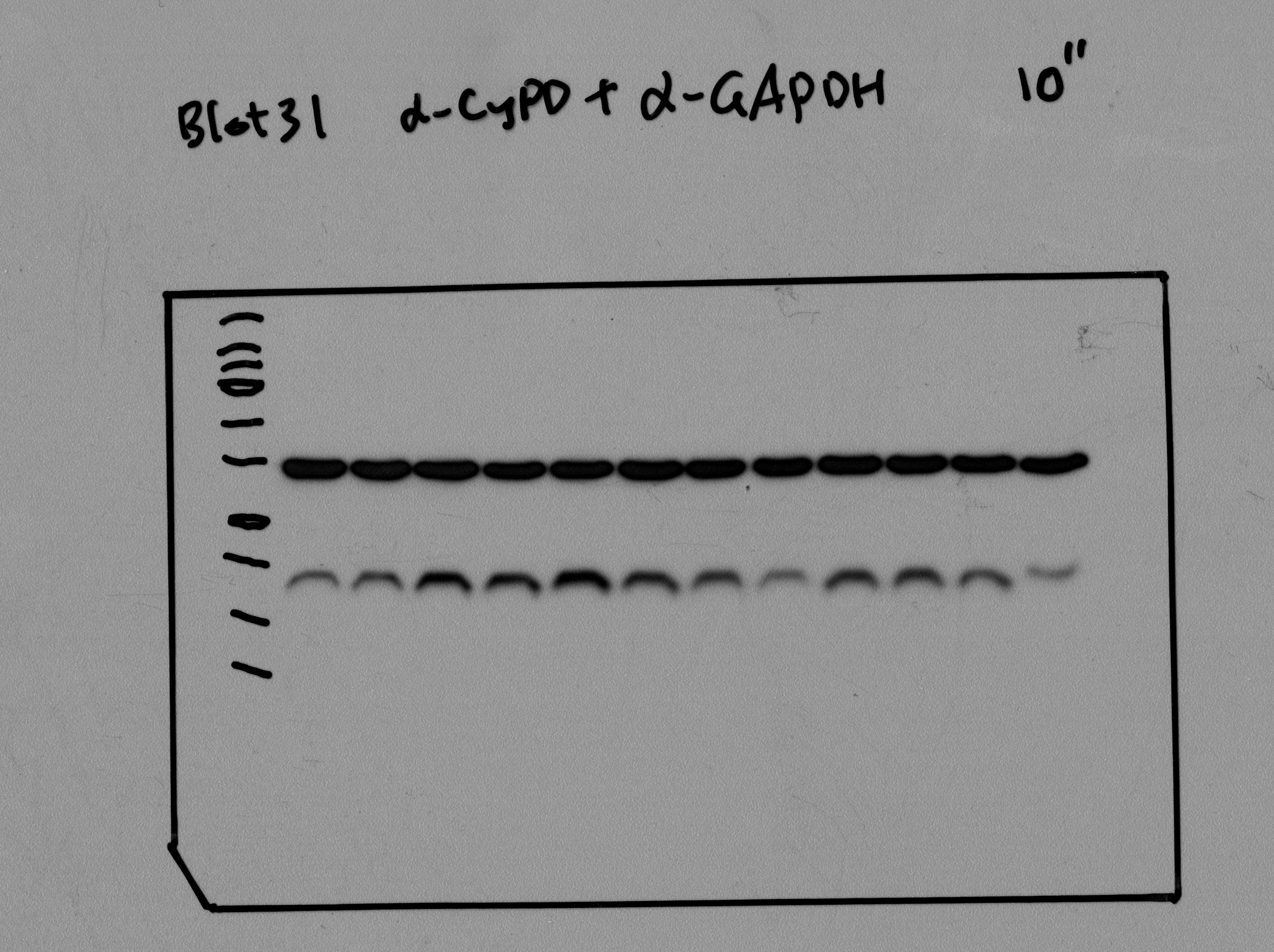

Supplement: Supplementary file 1 [file ijms-22-07412-s001.zip › Figure S2.png]
